# TNF-α and IFN-γ prestimulation enhances the therapeutic efficacy of human amniotic epithelial stem cells in chemotherapy-induced ovarian dysfunction

**DOI:** 10.1186/s41232-023-00309-y

**Published:** 2023-11-22

**Authors:** Yating Huang, Qiuwan Zhang, Wenjiao Cao, Qinyu Zhang, Lulu Wang, Dongmei Lai

**Affiliations:** 1 The International Peace Maternity and Child Health Hospital, School of Medicine, Shanghai Jiao Tong University, Shanghai, China; 2 Shanghai Key Laboratory of Embryo Original Diseases, 145, Guang-Yuan Road, Shanghai, 200030 China

**Keywords:** Human amniotic epithelial stem cells, Premature ovarian failure/insufficiency, Inflammatory cytokine, Prestimulation, Paracrine secretion, Oxidative damage

## Abstract

**Background:**

Exposure to a harsh ovarian microenvironment induced by chemotherapeutic agents seriously affects the remodeling of ovarian function and follicular development, leading to premature ovarian failure or insufficiency (POF/POI). For decades, the effectiveness of stem cell therapies in POI animal models has been intensively studied; however, strategies to enhance the therapeutic effect of stem cells remain challenging.

**Methods:**

In this study, we first observed the pathological changes of the ovaries at different time points during chemotherapy, including the number of follicles, granulosa cell proliferation, oxidative stress damage, ovarian fibrosis, and inflammatory reaction. Moreover, we investigated whether activated hAECs stimulated by the proinflammatory cytokines tumor necrosis factor-α (TNF-α) and interferon-γ (IFN-γ) were more effective than native hAECs in repairing ovarian injury induced by chemotherapy.

**Results:**

The inhibitory effect of chemotherapy drugs on ovarian granulosa cells (GCs) in growing follicles mainly occurred on day 3 after chemotherapy in a mouse model. Then, continued ovarian injury, including oxidative damage and cell death cascades, resulted in the depletion of follicular reserves and inflammation-related ovarian fibrosis. Cytokine array demonstrated that activated hAECs secreted high levels of paracrine cytokines related to extracellular matrix (ECM) remodeling, angiogenesis, and immunomodulation. An in vivo study showed that the engraftment rate of activated hAECs in damaged ovaries was higher than that of native hAECs. Furthermore, activated hAECs in damaged ovaries had significantly upregulated expression of the antioxidant proteins thioredoxin1/2. In addition, activated hAECs had increased numbers of mature follicles and ameliorated the ovarian microenvironment by promoting angiogenesis and reducing ovarian fibrosis.

**Conclusions:**

These results indicated that secondary ovarian damage induced by chemotherapy, including oxidative stress damage, chronic inflammatory response, and ovarian tissue fibrosis should be attended. Prestimulation with the proinflammatory factors TNF-α and IFN-γ could enhance the therapeutic efficacy of hAECs against chemotherapy-induced ovarian dysfunction, which may become a new feasible strategy to improve the therapeutic potential of hAECs in regenerative medicine.

**Graphical Abstract:**

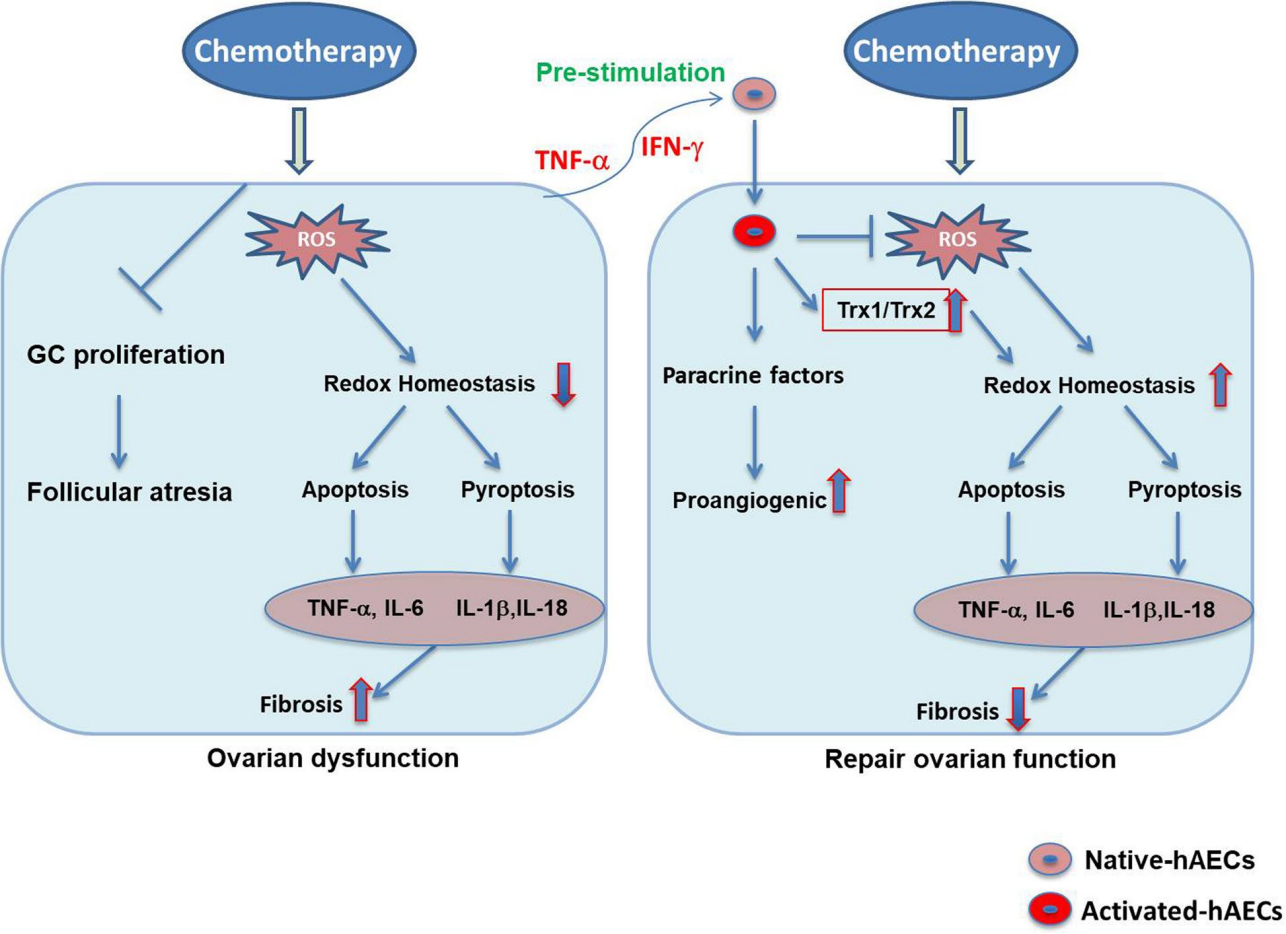

**Supplementary Information:**

The online version contains supplementary material available at 10.1186/s41232-023-00309-y.

## Background

Chemotherapeutic agents greatly improve the efficacy of cancer treatment and prolong the survival of cancer patients, but inevitably lead to reproductive toxicity in young female cancer survivors [1]. Previous histological studies on the human ovary have shown that chemotherapy could cause the depletion of follicular reserves and ovarian tissue fibrosis and ultimately lead to premature ovarian failure or insufficiency (POF/POI) [2]. Although some efforts have been made to determine the cause of chemotherapy-induced ovarian dysfunction, the underlying molecular mechanism remains unclear.

In animal studies, chemotherapy drugs exert negative effects on the ovary through distinct mechanisms, and DNA damage-induced cell apoptosis is considered to be the principal mechanism of irreversible decline in the ovarian reserve [3]. In addition to direct DNA damage, chemotherapy-induced oxidative stress is accompanied by increased production of reactive oxygen species (ROS) [4]. A clinical study reported that the level of malondialdehyde (MDA), which is a marker of oxidative damage, was significantly increased in patients receiving high-dose chemotherapy [5]. The increase in serum oxidative stress may be a promising indicator of the risk of primary ovarian insufficiency [6]. Oxidative stress-related mitochondrial dysfunction leads to apoptosis in ovarian cells, resulting in declines in ovarian function and the number and quality of oocytes [7]. In addition, oxidative stress could upregulate the expression of proinflammatory cytokines by activating a variety of transcription factors. Although inflammatory factors are indispensable in the reproductive process, excessive inflammatory reactions cause abnormal follicular development and ovarian fibrosis [8]. Women with the low and high tumor necrosis factor-alpha receptor 2s (TNFR2) levels were much more likely to have a risk of early menopause than those with medium levels of TNFR2 [9], suggesting that inflammation may be an important cause of ovarian dysfunction. Our previous research showed that chemotherapy drugs caused granulosa cell (GC) apoptosis and inflammatory reactions in the ovaries of mice [10]. However, the relationship between oxidative stress and chronic inflammation in the pathological process of chemotherapy-induced POF/POI has not been completely elucidated.

Stem cell therapy brings new hope for the treatment of diseases and the recovery of tissue function. Numerous studies have showed the repair potential of stem cells, which can differentiate into desired cell types, activate the endogenous response, promote angiogenesis, and improve the tissue microenvironment [11]. Human amniotic epithelial stem cells (hAECs) derived from placentas have several unique advantages over other stem cells, including no rejection, low proliferative potential due to a lack of telomerase expression, and the avoidance of ethical concerns [12]. Our previous studies have indicated that the transplantation of hAECs and hAEC derivatives (paracrine cytokines and exosomes) could effectively repair ovarian function and improve the fertility of mice in a chemotherapy-induced POF/POI model by homing and differentiating into ovarian GCs, as well as exerting proangiogenic and anti-inflammatory effects [10, 13, 14]. Although the application of stem cells has been shown to improve preclinical and clinical outcomes, there are still many challenges to overcome. The main limitation is the loss of cell viability and the reduction in repair ability after systemic or local transplantation [15]. Therefore, it is necessary to establish a new strategy to improve their therapeutic efficacy.

At present, researchers have proposed using prestimulation to strengthen the repair abilities of stem cells, in which stem cells are subjected to mild and transient stimulation before engraftment [16]. Modified models include genetic engineering, inducing differentiation into specific cell types, and activation with damaged signal molecules such as proinflammatory cytokines [17]. In several prestimulation models, the effect of proinflammatory cytokines on stem cells has been highlighted in mesenchymal stem cells (MSCs), and the expression of immunomodulatory proteins and the production of soluble factors were upregulated to improve MSC-mediated repair potential [18]. A recent study showed that hAECs stimulated with cytokines TNF-α and IFN-γ could alleviate dextran sulfate sodium (DDS)-induced colitis in mice through anti-inflammation and regulating Th17/Treg balance [19]. However, it is not clear whether prestimulation with proinflammatory factors could enhance the therapeutic potential of hAECs to repair ovarian function.

The main purpose of this study was to examine the roles of oxidative stress and chronic inflammation in chemotherapy-induced ovarian dysfunction. Moreover, we further investigated the paracrine ability of hAECs stimulated by TNF-α and INF-γ in vitro and evaluated the therapeutic effect of activated hAECs in a chemotherapy-induced ovarian dysfunction mouse model.

## Methods

### Isolation and culture of hAECs

Informed consent was obtained from healthy women who tested negative for HIV-I, hepatitis B, and hepatitis C prior to obtaining human placentas. Approval of the acquisition protocol was provided by the Institutional Ethics Committee of the International Peace Maternity and Child Health Hospital (IPMCH). The hAEC isolation method was described previously [10]. Isolated hAECs were seeded in 100 mm cell culture plates containing Dulbecco’s modified Eagle’s medium/nutrient mixture F-12 (DMEM/F12, Gibco, Grand Island, NY, USA) containing 10% fetal bovine serum (FBS, Gibco), 2 mM glutamine, penicillin (100 IU/mL; Gibco), streptomycin (100 μg/mL; Gibco), and epidermal growth factor (EGF, 10 ng/mL). Incubators were set at 37 °C and contained 5% CO_2_. Cells were collected for subsequent experiments when they reached 80–90% confluence.

### POF/POI model establishment

A total of 76 female C57BL/6 mice aged 7–8 weeks were obtained from the Shanghai Experimental Animal Center of the Chinese Academy of Sciences and reared at room temperature 25±2°C, relative humidity 55±5%, and a 12h-light/dark cycle. The mice were reared in an animal facility for 1 week before the experiment. The chemotherapy-treated group and Sham control group were divided randomly. Briefly, single doses of 30 mg/kg of busulfan (Bu, Sigma) and 120 mg/kg of cyclophosphamide (CTX, Sigma) were injected intraperitoneally in the chemotherapy-treated group (Cy, *n*=45) to establish the chemotherapy-induced POF/POI model as described previously [10]. Moreover, an equivalent volume of PBS was injected into mice in the sham-control group (Sham, *n*=31). All procedures were approved by the Institutional Animal Care and Use Committee of Shanghai standards and the National Research Council Guide for the Care and Use of Laboratory Animals. Efforts were made to alleviate animal suffering and use the fewest possible number of animals with restrictions in the study.

### Histological analysis

Mice in different groups were euthanized for further analysis. Ovaries were collected at different time points after chemotherapy for histological analysis. The tissues were immersed in Bouin’s liquid (containing 5% of acetic acid, 9% formaldehyde, and 0.9% of picric acid) at room temperature. Then, the ovaries were dehydrated and embedded in paraffin. The morphological structure of the ovary was evaluated under a light microscope with hematoxylin and eosin (HE)-stained slides. Follicle stage classification was performed according to previously defined criteria [13]. In brief, blind follicle counts were conducted by two independent researchers who examined every fifth section of the entire ovary. A primordial follicle refers to a single fusiform oocyte surrounded by GCs. A primary follicle indicates the unit of an oocyte surrounded by at least three cubic-shaped GCs. A secondary follicle is characterized by an oocyte surrounded by at least two layers of GCs with follicular cavity deficiency. Mature follicles (also called antral follicles) contain at least two layers of GCs with an evident follicular cavity.

### Biochemical assays

The levels of oxidative stress in the ovaries and serum of mice in the different treatment groups were measured. Biochemical analysis kits (Beyotime, Biotechnology, China) were used to measure MDA concentrations and antioxidant capacity according to the manufacturer’s instructions.

### Western blotting analysis

Sodium dodecyl sulfate-polyacrylamide gel electrophoresis (SDS-PAGE) was performed, and the separated proteins were then transferred into polyvinylidene difluoride (PVDF) membranes and blocked with skimmed 5% milk diluted with TBST (tris-buffered saline, 10 mM Tris-HCl pH 7.5, 150 mM NaCl, and 0.1% Tween-20) for 1 h at room temperature. Then, the membrane was incubated with the following primary antibodies at 4°C overnight: mouse anti-proliferating cell nuclear antigen (PCNA, 1:1000), rabbit anti-pAkt (1:1000), rabbit anti-Akt (1:1000), rabbit anti-p-Rps6 (1:1000), rabbit anti-Rps6 (1:1000), rabbit anti-pFoxo3a (1:1000), rabbit anti-Foxo3a (1:1000), rabbit anti-pPTEN (1:1000), rabbit anti-PTEN (1:1000), rabbit anti-Caspase 3 (1:1000), rabbit anti-Cleaved-Caspase 3 (1:1000), rabbit anti-Bcl2 (1:1000), rabbit anti-Bax (1:1000), rabbit anti-Caspase 1 (1:1000), rabbit anti-Cleaved-Caspase 1 (1:1000), rabbit anti-NLRP3 (1:1000), rabbit anti-AIM2 (1:1000), rabbit anti-ASC (1:1000), rabbit anti-Thioredoxin1/2 (1:1000), rabbit anti-PRDX1 (1:1000), rabbit anti-TXNIP (1:1000), rabbit anti-TRXR1 (1:1000), mouse anti-SOD2 (1:5000), anti-Tubulin (1:10,000), and anti-GAPDH (1:10,000). On the following day, the membranes were incubated with horseradish peroxidase-labeled secondary antibodies (1:3000, CST) for 1 h at room temperature. Protein expression was examined using the ECL method (Santa Cruz Biotechnology, Dallas. TX, USA). The relative intensity of the protein bands was quantified by digital densitometry using ImageJ software (National Institutes of Health, Bethesda, MD, USA). The levels of tubulin and GAPDH were used as internal standards.

### Enzyme-linked immunosorbent assay (ELISA)

Serum levels of TNF-α, interleukin (IL)-6, IL-10, CCL2, IL-1β, and IL-18 were measured by specific murine ELISA kits according to the manufacturer’s instructions. Serum samples were assayed in duplicate.

### Transmission electron microscopy (TEM)

Approximately 1 mm^3^ of fresh ovarian tissue was obtained and instantly fixed with 2.5% glutaraldehyde at room temperature. Ultrathin sections were prepared to observe mitochondrial ultrastructural morphology, and the sections were placed on copper grids and stained with uranyl acetate and lead citrate for evaluation by TEM.

### Sirius red staining

For Sirius red staining, after routine xylene dewaxing and graded ethanol hydration, paraffin ovarian sections were then stained with Picric acid-Sirius red (PSR) solution for 40 min at room temperature. The PSR solution was prepared with Sirius Red F3BA (Sigma-Aldrich) dissolved in a saturated aqueous solution of picric acid (Sigma-Aldrich) at 0.1% w/v. The immersion was followed by incubation with 0.5% glacial acetic acid and incubation with 0.05 M hydrochloric acid, in addition to four washes. The sections were further rapidly dehydrated in 100% ethanol after excess acidified water was carefully removed from the sections. For each independent experiment, all PSR staining took the same amount of time to minimize variations in staining intensity.

### Stimulation of hAECs with proinflammatory factors

hAECs were stimulated with proinflammatory cytokines for 24 h when they reached 60–70% confluence as described previously [19]. Briefly, the cells were treated with inflammatory factors TNF-α (10 ng/ml) plus IFN-γ (10 ng/ml) (all reagents from PeproTech).

### Cytokine array

To further investigate the changes in paracrine components from native and activated hAECs stimulated by TNF-α and IFN-γ, we used the methods described in a previous study [13]. First, a total of 2×10^6^ hAECs were cultured in 100-mm culture dishes until they reached 80% confluence and were then stimulated with TNF-α and IFN-γ. Twenty-four hours later, activated hAECs were washed with PBS to remove the cytokines and then cultured in an FBS-free medium for another 48 h. After the cells were cultured, conditioned medium from activated hAECs was collected and centrifuged at 300×g for 5 min to remove cell debris. Control conditioned medium from unstimulated hAECs (native hAECs) was generated in the same way except for the addition of cytokines to the culture dish. To measure the secretion levels of factors, a cytokine assay (AAH-BLG-1, Ray Biotech, Norcross, GA, USA) was performed.

### Tube formation assay

To study the proangiogenic effects of secretions from native and activated hAECs, conditioned medium from native hAECs (native-hAEC-CM) and conditioned medium from activated hAECs (activated-hAEC-CM) were collected. Human umbilical vein endothelial cells (hUVECs) were cultured in DMEM/F12 supplemented with 10% FBS. A total of 3×10^4^ hUVECs were seeded in the bottom chamber of a Matrigel-coated 24-well plate. Then, the culture medium of hUVECs was replaced with native-hAEC-CM or activated-hAEC-CM for 4 h to allow tube-like structure formation. The tube-like structures were observed under a light microscope, and the number of tubes and nodes was quantified by ImageJ software.

### Tracing transplanted hAECs

To observe the homing ability of grafted cells, native hAECs and activated hAECs were prelabeled with the fluorescent dye PKH26 (Sigma Aldrich, St. Louis, MO, USA) according to the manufacturer’s instructions. PKH26-labeled native hAECs and activated hAECs were microinjected into the damaged ovary (2×10^4^ cells, a volume of 20 μL) on day 7 after chemotherapy. At 1 week after transplantation, the ovaries were collected and frozen sections were prepared at a thickness of 7 μm. Then, the ovarian sections were stained with DAPI for 5 min at room temperature. The fluorescent signal of PKH26 in frozen ovarian sections was examined under an inverted fluorescence microscope.

### Cell transplantation

Native hAECs and activated hAECs were microinjected into the injured ovaries of chemotherapy-treated mice (2×10^4^ cells, a volume of 20 μl) on day 7 after chemotherapy. The mice in the chemotherapy control group were injected with an equivalent volume of PBS. The animals were sacrificed for subsequent experiments 1 and 4 weeks after cell transplantation.

### Measurement of ROS

The level of ROS in the ovary was measured according to the manufacturer’s instructions. Frozen ovarian sections were incubated with DCFH-DA for 10 min at 37 °C. After being washed, the fluorescence level was immediately examined under an inverted fluorescence microscope and photographed. The mean fluorescence intensity was analyzed using the ImageJ software.

### Immunofluorescence labeling

To assess macrophage infiltration in damaged ovaries, dual staining with CD68 and CD163 was performed. Sections were incubated with primary mouse monoclonal anti-CD68 antibody (1:1000; Abcam) and rabbit monoclonal anti-CD163 antibody (1:1000; Abcam) overnight at 4 °C after being dewaxed. After being washed with PBS, the sections were incubated with the corresponding secondary antibodies conjugated with Alexa Fluor 488 and 594 (1:3000; Cell Signaling Technology, CST). The fluorescent signals were captured and photographed with a TCS SP5 confocal laser scanning microscope (Leica).

### Immunohistochemical (IHC) staining

The ovarian sections were immersed in boiling sodium citrate solution for antigen retrieval, followed by routine dewaxing and hydration. The rest of the procedure was performed according to the instructions of the IHC staining kit (Abcam). Briefly, the slides were soaked in hydrogen peroxide solution and blocked with diluted goat serum, followed by overnight incubation with primary antibodies against Ki-67 (1:500, Abcam), DNA/RNA damage marker (1:1000, CST), and CD34 (1:1000, CST). The next day, the sections were washed and successively incubated with biotinylated anti-mouse/rabbit IgG, streptavidin peroxidase, and DAB chromogen solution. Some slides were counterstained with hematoxylin. The negative control samples were subjected to identical treatments, except for primary antibodies, and exhibited no specific staining. The number of microvessels was calculated by counting the number of CD34-positive vessel-like structures in four randomly selected fields in each section with ImageJ software.

### Statistical analysis

The data was presented as the mean±standard error of the mean (SEM). For statistical analysis, differences among the different treatment groups were analyzed with one-way ANOVA followed by the Bonferroni post hoc test. All statistical analyses were performed using GraphPad Prism Software (San Diego, CA, USA).

## Results

### Chemotherapy-induced growth inhibition in ovarian GCs occurred in the early stage in vivo

**Fig. 1:**
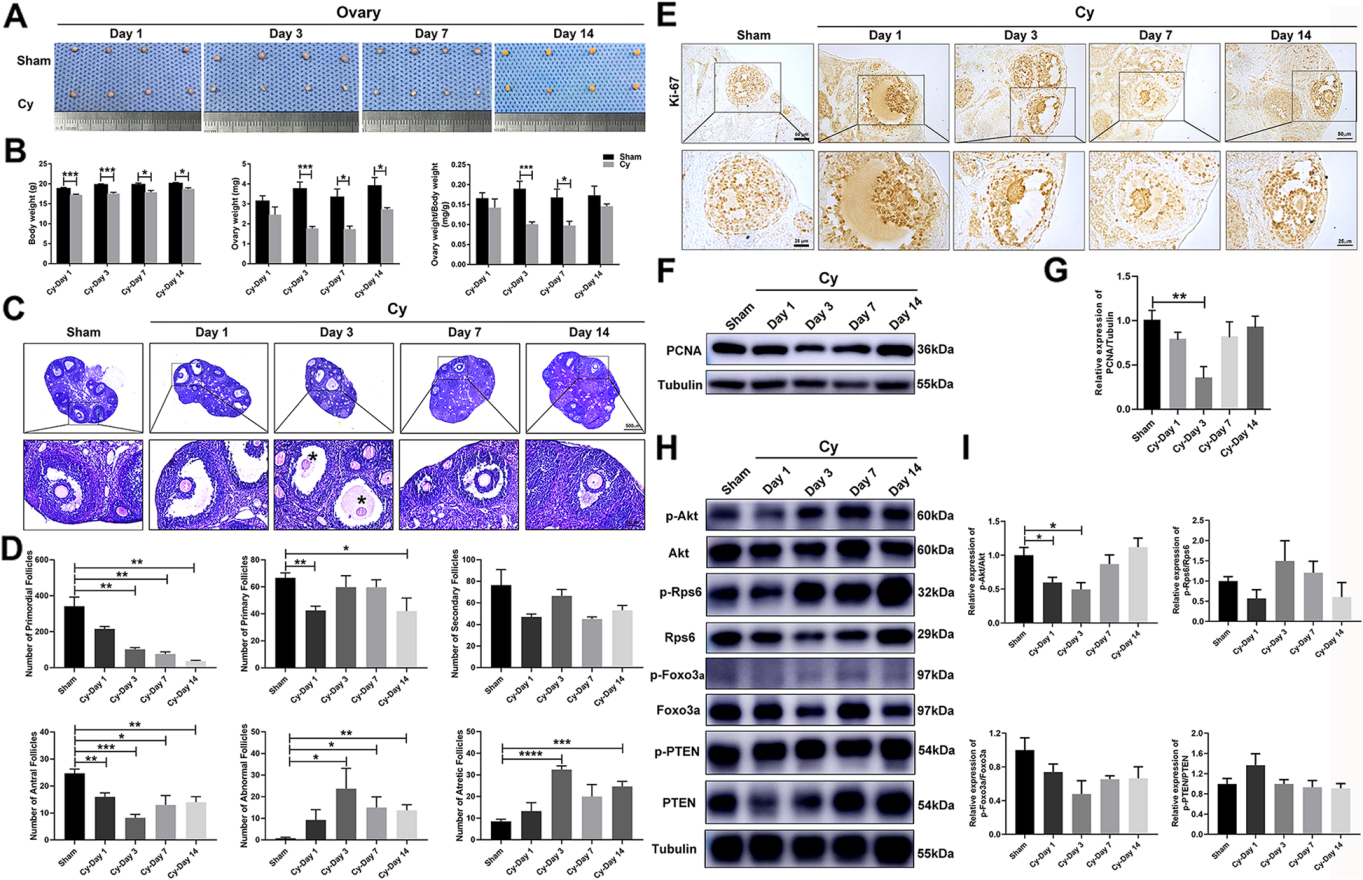
Effect of chemotherapy drugs on ovaries at different time points. **A** Images showing the ovaries in the sham and chemotherapy (Cy)-treated groups on days 1, 3, 7, and 14. **B** The columns showed the body weight and ovarian weight of mice in the sham and Cy-treated groups. **C** Images showing HE staining of ovarian sections from the sham and Cy-treated groups. The asterisk represented a naked oocyte. Scale bars, 500 μm and 100 μm. **D** The columns showed the number of follicles at different stages, including primordial follicles, primary follicles, secondary follicles, and antral follicles, as well as the number of abnormal follicles and atretic follicles. **E** Representative images showing Ki-67 expression in ovarian sections from the sham and Cy-treated groups. Scale bars, 50 μm and 25 μm. **F**, **G** The protein expression of PCNA in ovaries was examined and analyzed at different time points after chemotherapy. **H**, **I** The expression of follicular activation-related proteins was examined and analyzed at different time points after chemotherapy. *N*=4 per group. The data were presented as the mean±SEM. *P<0.05, **P<0.01, ***P<0.005, and ****P<0.001. Cy, chemotherapy; PCNA, proliferating cell nuclear antigen

Our previous study demonstrated that a single injection of high-dose chemotherapy drugs could induce ovarian injury, ultimately leading to POF/POI in mice [10]; however, the underlying mechanisms are still unknown. In the current study, we collected ovarian tissues at different time points after chemotherapy (Cy), as shown in Fig. 1A. The results showed that the ovarian index (ovary weight/body weight) of mice decreased significantly on days 3 and 7 after chemotherapy (Fig. 1B, *P*<0.05). Morphological analysis showed that the GC layer of antral follicles in the Cy-treated group was thinner than that in the sham group. Moreover, naked oocytes, as typical features, were present in antral follicles (abnormal follicles) on day 3 after chemotherapy (Fig. 1C). The number of primordial follicles and antral follicles decreased gradually after chemotherapy; however, the number of abnormal follicles and atresia follicles in damaged ovaries began to increase from day 3 after chemotherapy (Fig. 1D, *P*<0.05).

We further examined ovarian cell proliferation at different time points after chemotherapy by immunostaining of the proliferation marker Ki-67. The majority of Ki-67-positive cells were GCs in the antral follicles in the sham group; however, the proliferation of ovarian GCs was inhibited after chemotherapy (Fig. 1E). Western blotting analysis showed that the expression of proliferation marker (proliferating cell nuclear antigen, PCNA) in damaged ovaries decreased significantly on day 3 after chemotherapy (Fig. 1F-G, *P*<0.05). A previous study reported that the mechanism in chemotherapy-induced loss of ovarian reserve involved accelerated activation of primordial follicles via the PI3K/Akt pathway [20]; therefore, we further examined the expression of pathway-related proteins in Cy-treated ovaries by western blotting. The results showed that the protein expression of pAkt/Akt in damaged ovaries was significantly reduced on days 1 and 3 after chemotherapy; however, there were no differences on days 7 and 14 after chemotherapy (Fig. 1H–I, *P*<0.05).

These results demonstrated that the inhibitory effect of chemotherapy drugs on ovarian GCs mainly occurred in the early stage. Although the proliferation of GCs and activation of primordial follicles gradually recovered in the later stage of chemotherapy, the number of follicles in damaged ovaries continued to decrease.

### Chemotherapy caused oxidative damage and mitochondrial dysfunction in the ovary

**Fig. 2:**
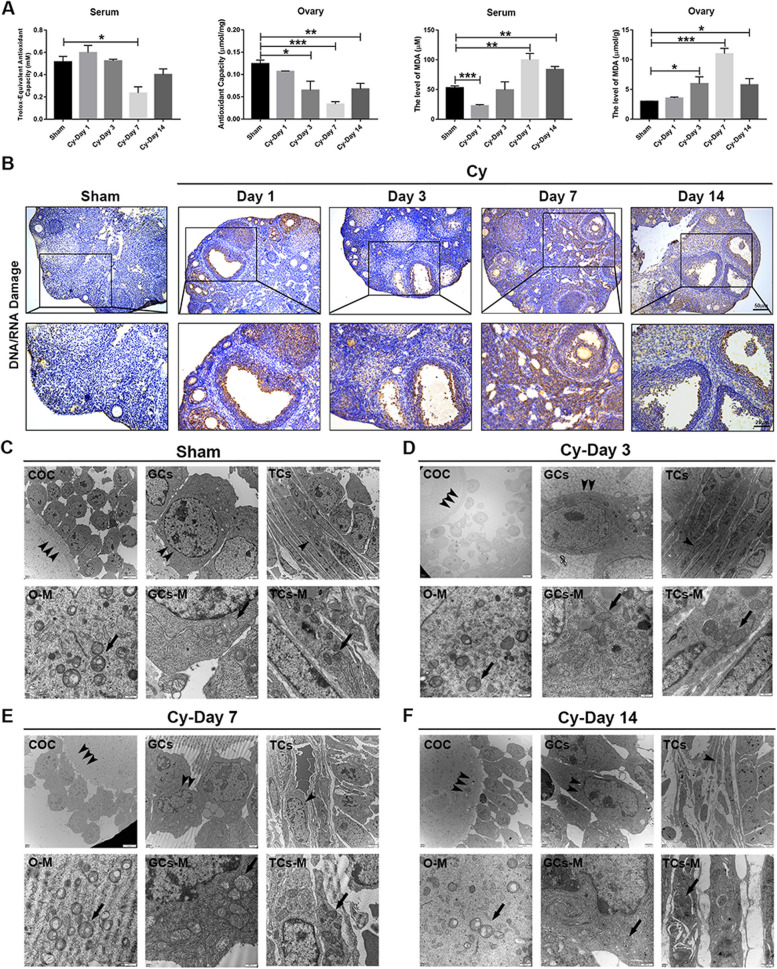
Effect of chemotherapy on oxidative damage and mitochondrial dysfunction in the ovary. **A** Determination of antioxidant capacity and the level of MDA in serum and ovaries from the different treatment groups. **B** IHC staining was performed to examine the expression of DNA/RNA damage markers in ovaries. Scale bars, 50 μm and 25 μm. **C**–**F** The ultrastructure and mitochondrial morphology of antral follicles in the different groups were examined by TEM. Abnormal mitochondria were swollen with evidence of severely disrupted cristae, indicated by black arrows. Scale bars, 5 μm, 1 μm, 2 μm, and 500 nm. *N*=4 per group. The data were presented as the mean±SEM. *P<0.05, **P<0.01, and ***P<0.005. COC, cumulus-oocyte complex; GCs, granulosa cells; TCs, thecal cells; O-M, oocyte-mitochondrial; GCs-M, granulosa cell-mitochondrial; TCs-M, thecal cells-mitochondrial

Chemotherapy induces DNA damage and oxidative stress by altering the redox balance [21]. To further elucidate the underlying mechanism of continued ovarian injury induced by chemotherapy, we measured the antioxidant capacity and the level of oxidative marker MDA in serum and ovaries in the different groups. Compared with that in the sham group, the serum antioxidant capacity decreased significantly on day 7, while the ovarian antioxidant capacity decreased dramatically on days 3, 7, and 14 after chemotherapy. The level of MDA in the serum and ovaries of mice in the Cy-treated group was higher than that in the sham group (Fig. 2A, *P*<0.05). Furthermore, the expression of oxidative stress-induced DNA/RNA damage markers was detected in the GCs of antral follicles in the ovaries of mice after chemotherapy (Fig. 2B).

To further observe follicular development in chemotherapy-damaged ovaries, cellular ultrastructure was examined by TEM. The results showed that the ultrastructure of the cumulus-oocyte complex (COC), GCs, and thecal cells (TCs) in the antral follicles of damaged ovaries changed evidently. Compared with that in the sham group, the connection between cumulus GCs and oocytes in the COC was loose, the nucleus of cumulus GCs shrank, and the arrangement of TCs was disordered in the Cy-treated group. Moreover, among the three types of cells, the mitochondria of GCs were swollen and had fragmented cristae, as shown in Fig. 2C–F. These results indicated that chemotherapy could lead to persistent oxidative damage in the ovary and mitochondrial dysfunction in GCs of antral follicles.

### Chemotherapy-induced cell death and inflammation-related fibrosis in the ovary

**Fig. 3:**
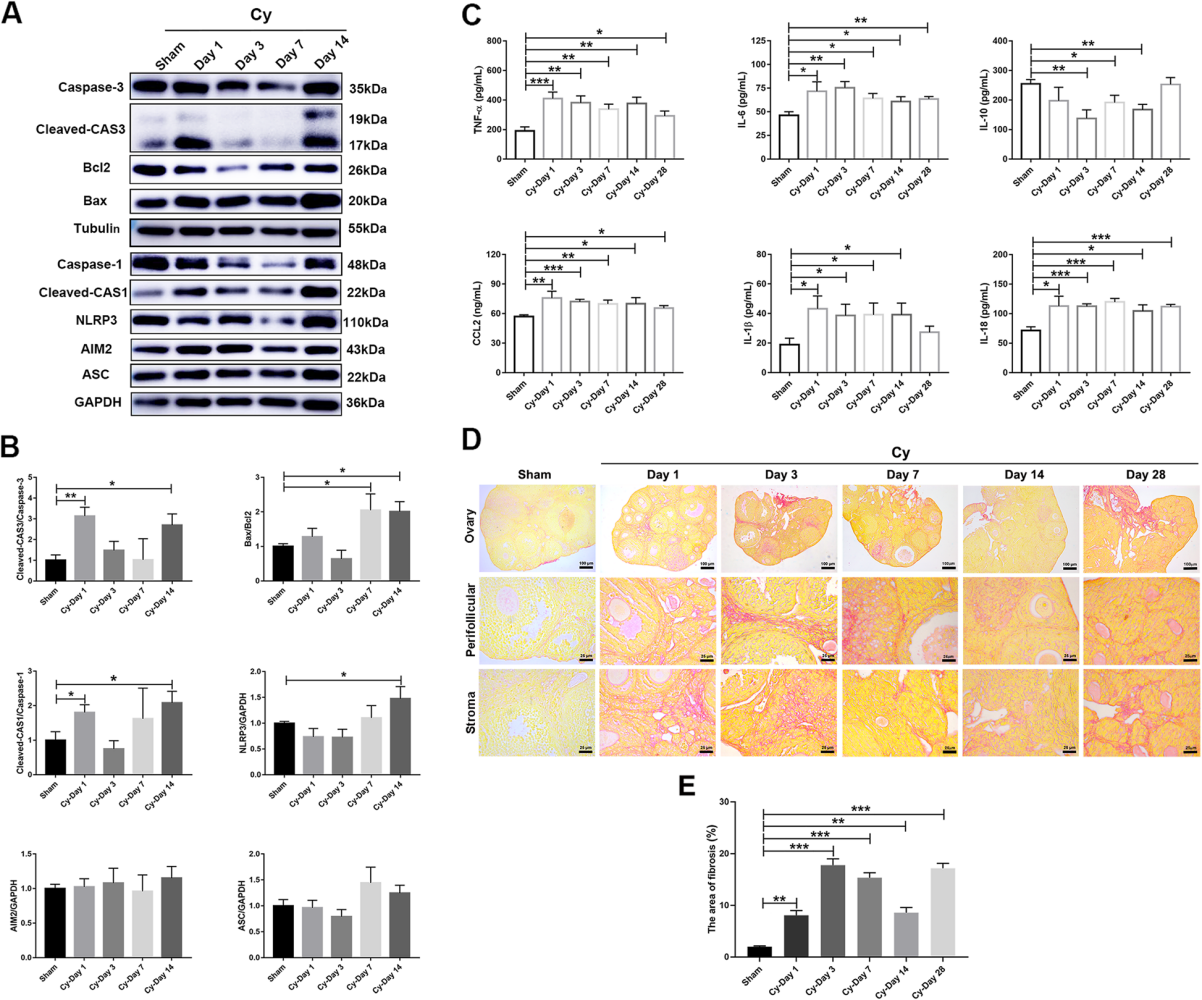
Effect of chemotherapy on cell death and inflammation-related fibrosis in the ovary. **A** Western blotting was used to examine the expression of apoptosis- and pyroptosis-related proteins in ovaries in the different treatment groups. **B** The columns showed that the expression of cleaved-CAS3, Bax/Bcl2, NLRP3, and cleaved-CAS1 significantly increased after chemotherapy. **C** ELISA was used to measure the level of inflammation-related cytokines in the serum of mice in the different treatment groups. **D** Representative images showing PSR-stained ovarian sections in the different groups. Scale bars, 100 μm and 25 μm. **E** The column displayed the area of fibrosis (PSR-positive staining) in ovaries in the different treatment groups. *N*=4 per group. The data were presented as the mean±SEM. *P<0.05, **P<0.01, and ***P<0.005

To evaluate cell death induced by oxidative damage in the ovary, the expression of apoptosis- and pyroptosis-related proteins in ovaries was determined by western blotting. The results showed that the expression of Cleaved-Caspase 3 (CAS3) increased significantly on day 1; however, there were no differences on days 3 and 7 after chemotherapy. Notably, the expression of Cleaved-CAS3 and Bax/Bcl2 increased again on day 14 in the Cy-treated groups. Moreover, the expression of the pyroptosis-related protein Cleaved-Caspase 1 (CAS1) in damaged ovaries increased significantly on day 1, and Cleaved-CAS1 and NLRP3 increased again on day 14 after chemotherapy (Fig. 3A, B, *P*<0.05).

Cell death can also induce inflammatory responses leading to persistent inflammation [22]; thus, we measured serum levels of inflammatory factors at different time points after chemotherapy. The results showed that the levels of proinflammatory factors, including TNF-α, IL-6, CCL2, IL-1β, and IL-18 in the Cy-treated groups increased significantly; however, the level of the anti-inflammatory factor IL-10 decreased significantly (Fig. 3C, *P*<0.05). The accumulation of inflammatory factors causes collagen deposition and ovarian fibrosis, which can be measured by PSR staining [23]. Morphological analysis showed that the area of fibrosis in the Cy-treated groups was higher than those that in the sham group (Fig. 3D, E, *P*<0.05). These data suggested that chemotherapy could lead to ovarian cell apoptosis and pyroptosis, while systemic chronic inflammation resulted in ovarian fibrosis.

### Inflammatory factors TNF-α and IFN-γ costimulation increased paracrine secretion of hAECs

**Fig. 4:**
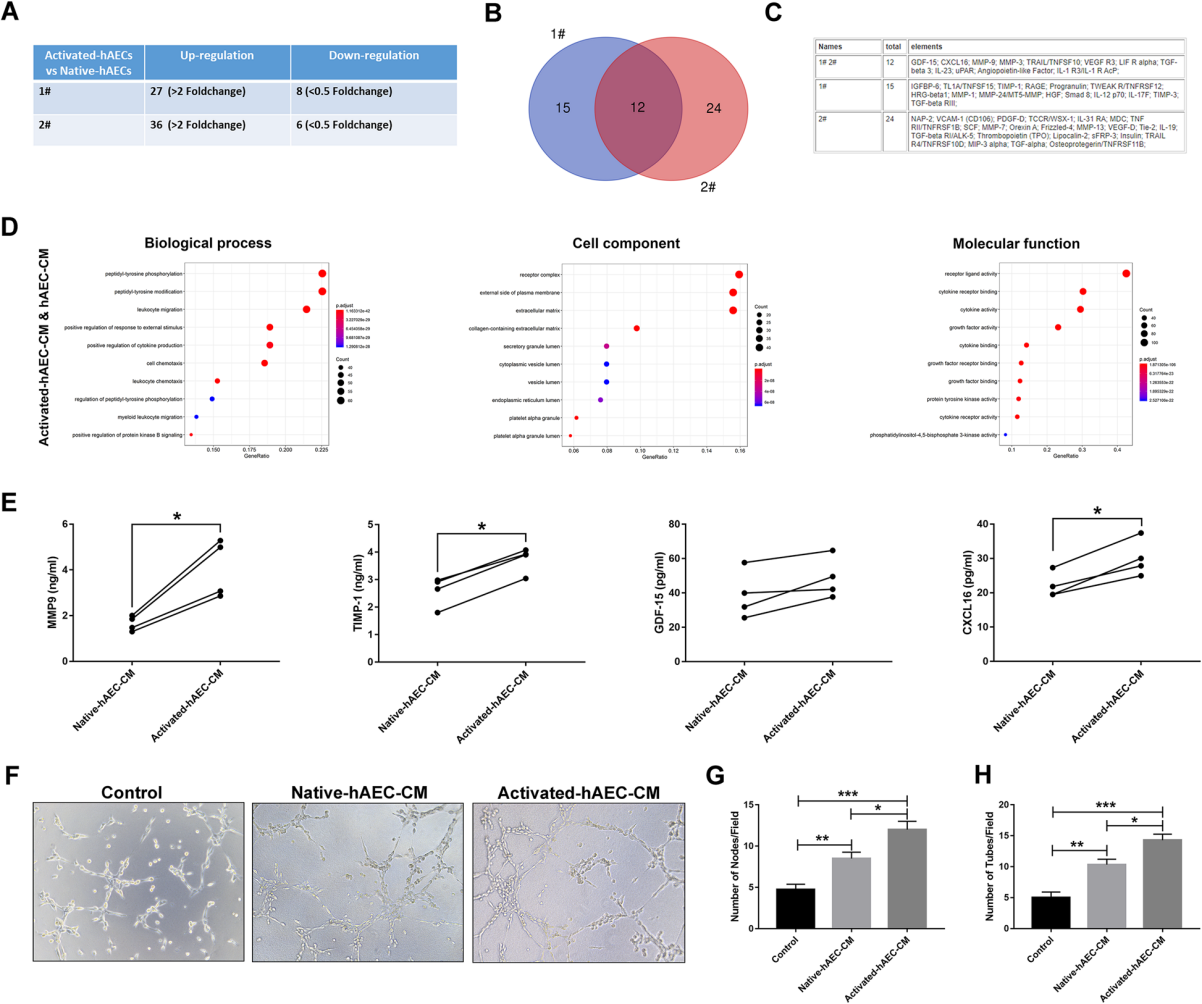
Effect of TNF-α and IFN-γ costimulation on the characteristics of hAECs. **A** The table shows the number of differentially expressed cytokines in the conditioned medium of native hAECs (native-hAEC-CM) and activated hAECs (activated-hAEC-CM) from donors #1 and #2. **B** Venn diagram showing the overlap of upregulated differentially expressed cytokines between donors #1 and #2. **C** All differentially upregulated cytokines were listed in the Table. **D** GO analysis of the biological process, cellular component, and molecular function categories of the upregulated cytokines. **E** ELISA was used to verify the levels of important cytokines in native-hAEC-CM and activated-hAEC-CM. **F** Representative images of the tube formation assay results in native-hAEC-CM and activated-hAEC-CM treatment groups. **G**, **H** Quantitative analysis of tube formation was conducted by counting tubes and nodes. The data were presented as the mean±SEM. *P<0.05, **P<0.01, and ***P<0.005

Our previous study has demonstrated that hAECs can secrete a variety of bioactive cytokines, which play important roles in promoting angiogenesis and inhibiting inflammation. Thus, a cytokine array was performed to examine secreted cytokines in the conditioned medium of native hAECs (native-hAEC-CM) and activated hAECs (activated-hAEC-CM). The results indicated that 27 proteins (#1) and 36 proteins (#2) were differentially expressed more than 2-fold in activated hAEC-CM compared with native hAEC-CM (Fig. 4A, *P*<0.05). The table showed 12 upregulation proteins in Group #1 and #2, including tissue remodeling proteins (MMP-9 and MMP-3), proangiogenic factors (angiopoietin-like factor and CXCL16), and immunomodulatory proteins (GDF-15 and TGF-β) (Fig. 4B, C). GO analysis showed that the upregulated secreted proteins were mainly enriched in cytokine-cytokine receptor interactions, the JAK-STAT signaling pathway, the PI3K-Akt signaling pathway, the MAPK signaling pathway, the IL-17 signaling pathway, and the cytokine receptor binding (Fig. 4D). The ELISA results further confirmed that the concentrations of MMP-9, TIMP-1, and CXCL16 in activated hAEC-CM were significantly higher than those in native hAEC-CM (Fig. 4E, *P*<0.05). In addition, the tube formation assay showed that activated hAEC-CM significantly increased the tube formation of hUVECs compared with native hAEC-CM (Fig. 4F–H, *P*<0.05). These results suggested that TNF-α and IFN-γ costimulation changed paracrine secretion of hAECs.

### Activated hAECs exhibited a high retention rate in injured ovaries and upregulated expression of antioxidant proteins

**Fig. 5:**
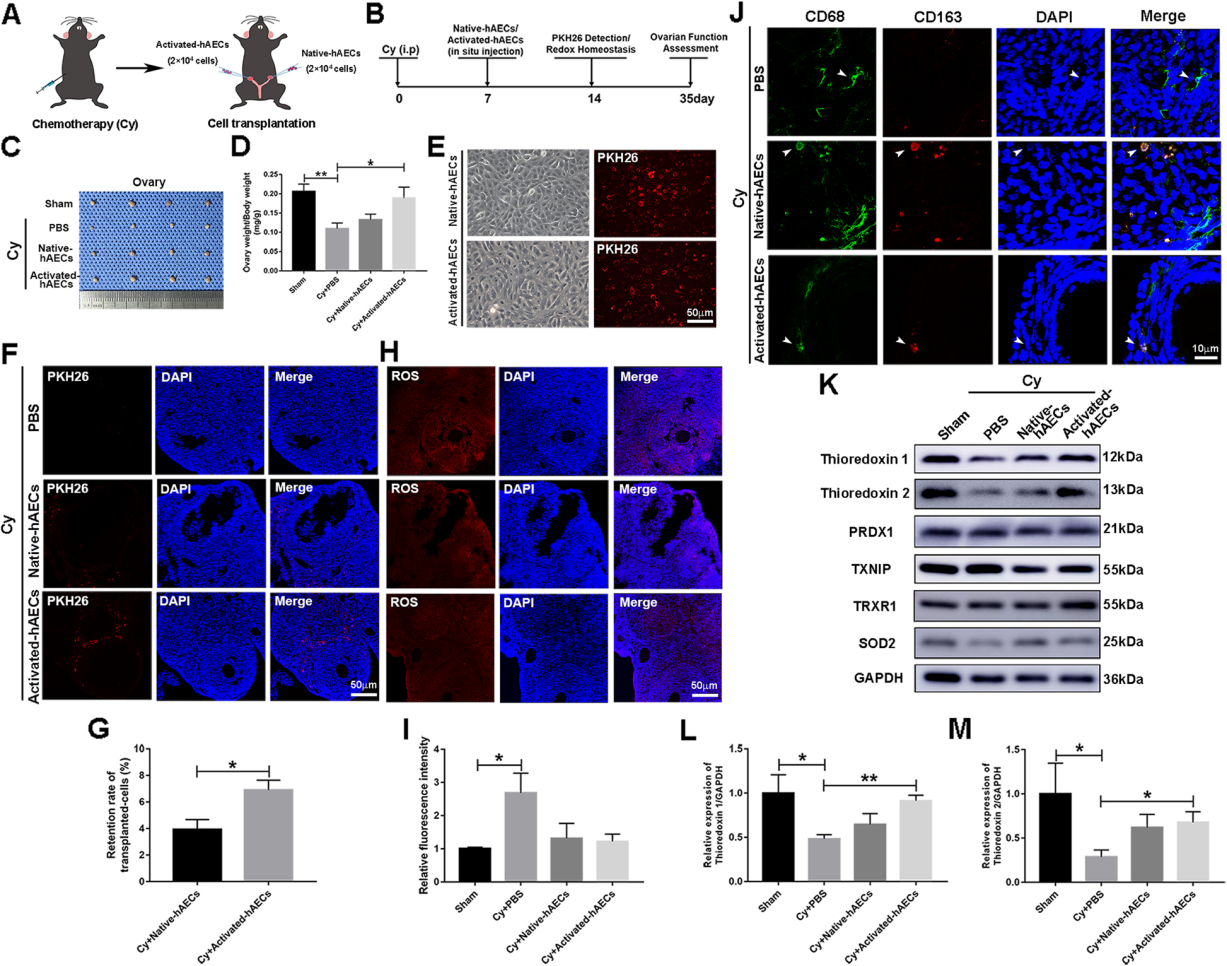
Effect of native hAECs and activated hAECs transplantation on injured ovaries. **A**, **B** Schematic diagrams showing the design of the animal experiment. **C** Representative images of ovaries in the different treatment groups. **D** The column showed the ovarian index (ovarian weight/body weight) of mice in the different treatment groups. **E** Native and activated hAECs were labeled with the fluorescent dye PKH26. Scale bar, 50 μm. **F** The red fluorescent signals in ovarian sections were observed under a fluorescence microscope. Scale bar, 50 μm. **G** The column showed the retention rate of transplanted native and activated hAECs in damaged ovaries. **H** The level of ROS in damaged ovaries was examined by immunofluorescent staining. Scale bar, 50 μm. **I** The column showed the level of ROS in ovaries after native and activated hAEC transplantation. **J** Double immunostaining of CD68 and CD163 in ovaries in the different groups. Scale bar, 10 μm. **K** Western blotting was performed to examine the expression of redox homeostasis-related proteins in ovaries. **L**, **M** The columns showed that activated hAEC transplantation significantly upregulated the expression of thioredoxin1/2 in damaged ovaries. *N*=4 per group. The data were presented as the mean±SEM. *P<0.05 and **P<0.01

To further study the therapeutic potential of activated hAECs in chemotherapy-induced ovarian dysfunction, we transplanted native hAECs and activated hAECs into the left and right ovaries, respectively, and assessed ovarian function, as shown in Fig. 5A, B. On day 7 after transplantation, we observed that activated hAEC transplantation significantly increased the ovarian index compared with that of the PBS group after chemotherapy (Fig. 5C, D, *P*<0.05). To observe the tissue retention rate of native hAECs and activated hAECs, the cells were prelabeled with fluorescent dye PKH26 before transplantation (Fig. 5E). The results showed that the red fluorescent signal of PKH26 was observed in the ovarian interstitial area, but not follicles on day 7 after transplantation (Fig. 5F). Compared with native hAEC transplantation, there were more red fluorescence signals in the damaged ovaries of the activated hAEC transplantation group (Fig. 5G, *P*<0.05). Furthermore, native hAECs and activated hAEC transplantation partially attenuated the increase in ROS induced by chemotherapy (Fig. 5H–I, *P*<0.05). Moreover, macrophage (CD68^+^) expressed M2 macrophage marker (CD163^+^) in damaged ovaries in the native and activated hAEC transplantation groups (Fig. 5J). In addition, the expression of redox homeostasis-related proteins in ovaries was further examined by western blotting. The results showed that activated hAEC transplantation significantly upregulated the expression of the antioxidant proteins thioredoxin1/2 in damaged ovaries (Fig. 5K–M, *P*<0.05). These results suggested that activated hAECs exhibited a high retention rate and upregulated the expression of antioxidant proteins in injured ovaries.

### Activated hAECs promoted follicular development by promoting angiogenesis and inhibiting fibrosis in injured ovaries

**Fig. 6:**
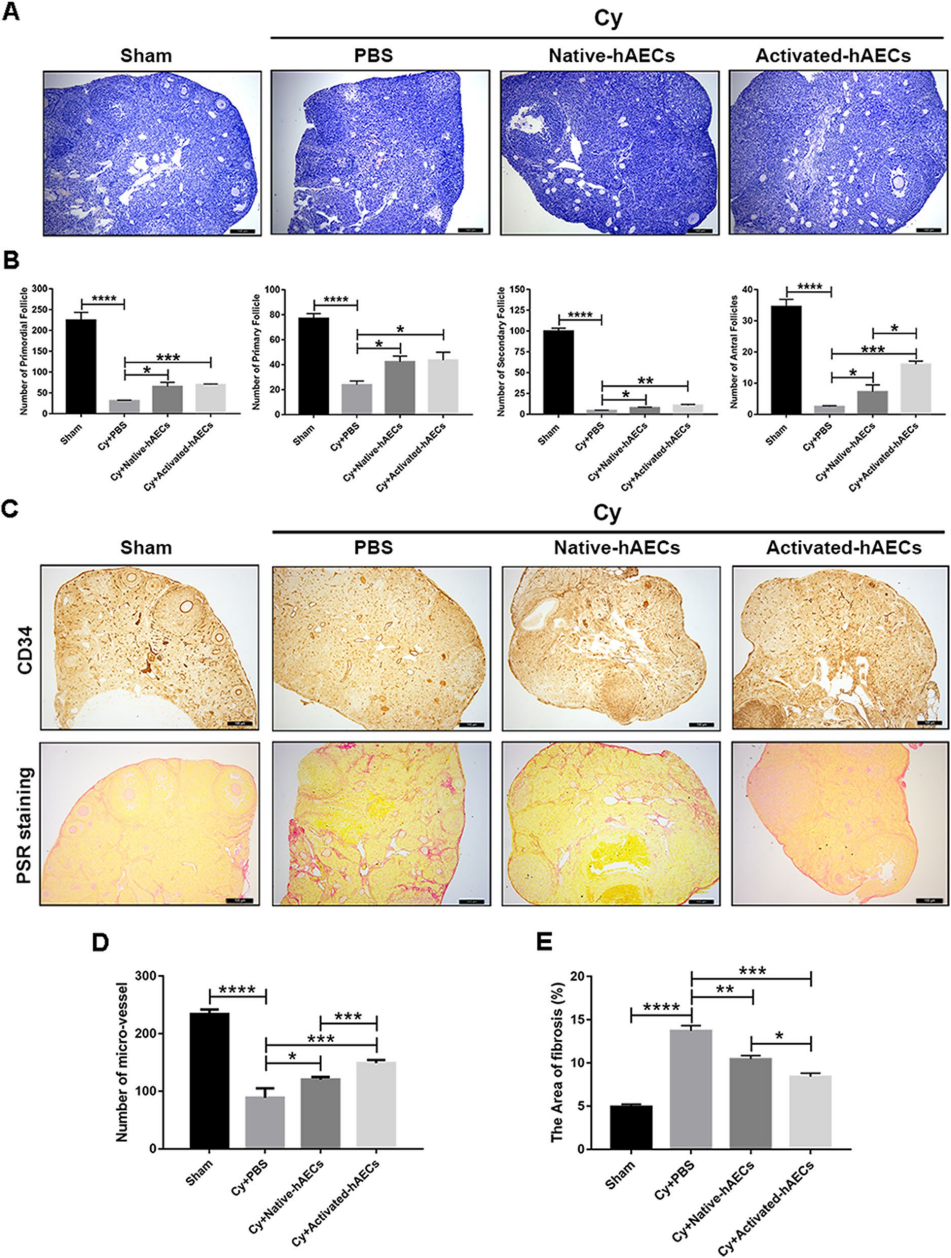
Effect of native hAECs and activated hAEC transplantation on follicular development and the ovarian microenvironment. **A** Representative images of HE-stained ovarian sections in the different treatment groups. Scale bar, 100 μm. **B** The columns showed the number of primordial, primary, secondary, and antral follicles in the ovaries of mice in the different treatment groups. **C** Representative images showing the expression of CD34 and PSR staining in ovarian sections in the different treatment groups. Scale bar, 100 μm. **D** The column showed the number of microvessels in ovaries in the different treatment groups. **E** The column showed the area of fibrosis (PSR staining) in ovaries in the different treatment groups. *N*=4 per group. The data were presented as the mean±SEM. *P<0.05, **P<0.01, ***P<0.005, and ****P<0.001

To investigate the therapeutic effect of activated hAECs in injured ovaries, follicular development in the different treatment groups was evaluated 1 month after cell transplantation. Morphological analysis showed a decrease in follicles in Cy-treated ovaries at different development stages compared with that in the sham group. In the native and activated hAEC transplantation groups, developing follicles were observed in damaged ovaries (Fig. 6A). The number of primordial, primary, secondary, and antral follicles in the native hAEC and activated hAEC transplantation groups was higher than in the PBS treatment group after chemotherapy. Notably, activated hAECs significantly increased the number of mature follicles in damaged ovaries compared with native hAEC transplantation (Fig. 6B, *P*<0.05). Ovarian function and follicular development are influenced by the ovarian microenvironment, including angiogenesis and vascular function [24] and the degree of fibrosis [25]. Ovarian angiogenesis and fibrosis in the different treatment groups were assessed by immunostaining for CD34 and PSR staining. The results showed that native and activated hAEC transplantation increased the number of microvessels (CD34 positive) and decreased ovarian fibrosis induced by chemotherapy. Intriguingly, we found that activated hAEC transplantation exerted a better effect on the expression of CD34 and the level of ovarian fibrosis in injured ovaries than native hAEC transplantation (Fig. 6C–E, *P*<0.05). These results indicated that activated hAEC transplantation had therapeutic potential in promoting follicular development by improving angiogenesis and reducing ovarian fibrosis in a chemotherapy-induced POF mouse model.

## Discussion

As the survival rates of tumor patients increase each year and the incidence of cancer tends to occur in younger individuals, an increasing number of female patients suffer from reproductive toxicity caused by chemotherapy, which is accompanied by amenorrhea, early menopause, and decreased natural pregnancy and live birth rates, which seriously affect the physical and mental health of patients [26]. The underlying mechanisms of chemotherapy-induced ovarian dysfunction have been widely studied, including excessive activation of primordial follicles and impairment of follicular maturation; however, less attention has been given to persistent ovarian injury in the late stage of chemotherapy.

Our previous study indicated that a single intraperitoneal injection of high-dose cyclophosphamide and busulfan could induce slow depletion of the ovarian reserve in mice, which was accompanied by apoptosis of GCs and acute vascular injury [10]. At different time points after chemotherapy, we observed that chemotherapy induced a gradual decrease in the follicle reserve in the ovaries of mice, according to the follicle counts. Moreover, the inhibitory effect of chemotherapy on the proliferation of ovarian GCs mainly occurred in the early stage of chemotherapy. A study indicated that chemotherapy drugs lead to the production of ROS and mitochondrial damage [27], and the imbalance in redox homeostasis in the ovarian microenvironment has been a major underlying cause of ovarian function impairment [28]. In the current study, we observed that exposure to chemotherapeutic agents disrupted the balance of the redox system and induced the accumulation of abnormal mitochondrial morphology in GCs in antral follicles. In addition to ovarian cell apoptosis, pyroptosis is another form of chemotherapy-induced cell death, which provides a new molecular target for inhibiting chemotherapy-induced ovarian injury.

In a chemotherapy-POF/POI mouse model, we have elaborated on the ovarian repair effect and underlying molecular mechanism of hAECs [10, 13, 14]. However, how to enhance hAEC-mediated repair needs to be further elucidated. Inflammation is an important pathological manifestation of tissue damage that affects the functional remodeling of tissues and organs [29]. Moreover, prestimulation with inflammatory factors in vitro could be used to strengthen the repair capacity of stem cells, including enhancing the expression of important molecules and enzymes, which are important for the repair outcomes of the stem cell transplantation [15]. In previous studies, we identified some important bioactive cytokines secreted by hAECs and demonstrated the effect of these cytokines on ovarian GCs, vascular endothelial cells, and macrophages [10, 13, 14]. A study reported that the effect of hAECs on angiogenesis could be affected by inflammation [30]. In this study, we found that TNF-α and IFN-γ prestimulation significantly increased the production of paracrine cytokines by hAECs, including the proangiogenic factors and matrix metalloproteinases (MMPs). In the chemotherapy-induced POF/POI mouse model, we observed that the retention rate of activated hAECs after transplantation was significantly higher than that of native hAECs in damaged ovaries. Moreover, an in vivo study demonstrated that activated hAEC transplantation significantly promoted angiogenesis in damaged ovaries. This beneficial effect may involve inflammatory factors stimulating the production of proangiogenic factors by hAECs. MMPs are tissue-remodeling enzymes that process various biological molecules. Studies have shown that MMP2 and MMP9 are produced by hAECs and play important roles in inhibiting fibrosis and promoting ECM remodeling in liver injury and fibrosis models [31]. Furthermore, MMP9 had anti-inflammatory effects and protected osteoblasts against LPS-induced inflammation [32]. Among the upregulated secretion factors, MMP9 was upregulated approximately 5-fold in activated hAECs, which could contribute to the attenuation of ovarian fibrosis induced by chemotherapy.

There are several limitations to the present study. First, we described persistent and secondary ovarian damage induced by chemotherapy, including an imbalance in oxidative stress, cell cascade death, and chronic inflammation. However, the inner relationships among these pathological changes have not been clarified. Second, prestimulation with inflammatory factors TNF-α and IFN-γ greatly changed the paracrine secretion characteristics of hAECs; however, the biological function of these high levels of cytokines requires further validation in animal models. Third, we observed that TNF-α and IFN-γ prestimulation enhanced the effectiveness of hAECs in repairing ovarian function, but the mechanistic investigation was not in-depth. In the future, we will continue to carry out in-depth studies to solve these issues.

## Conclusions

Our study revealed that the inhibitory effect of chemotherapeutic agents on the proliferation of ovarian GCs mainly occurred in the early stage of chemotherapy, but sustained ovarian damage affected follicular development and accelerated ovarian fibrosis. Furthermore, we found that appropriate prestimulation with proinflammatory factors TNF-α and IFN-γ could increase the production of paracrine cytokines by hAECs. In chemotherapy-induced POF/POI mice, activated hAECs exhibited a better retention rate in the damaged ovary and had increased expression of antioxidant proteins in ovaries. Moreover, activated hAECs significantly promoted the development of mature follicles by promoting angiogenesis and reducing ovarian fibrosis compared with native hAECs. Therefore, TNF-α and IFN-γ prestimulation may be a promising strategy to enhance the therapeutic strategy for the use of hAECs in regenerative medicine.

### Supplementary Information

**Additional file 1.**

## Data Availability

All the raw datasets generated during the current study are available from the corresponding author on reasonable request.

## Abbreviations

POF/POI: Premature ovarian failure/insufficiency
hAECs: Human amniotic epithelial stem cells
TNF-α: Tumor necrosis factor-α
IFN-γ: Interferon-γ
GCs: Granulosa cells
ECM: Extracellular matrix
ROS: Reactive oxygen species
MDA: Malondialdehyde
TNFR2: Tumor necrosis factor-alpha receptor 2s
MSCs: Mesenchymal stem cells
HE: Hematoxylin and eosin
hUVECs: Human umbilical vein endothelial cells
PSR: Picric acid-Sirius red
Cy: Chemotherapy
COC: Cumulus-oocyte complex
TCs: Thecal cells
