# Supplementary material for: TNF-α and IFN-γ prestimulation enhances the therapeutic efficacy of human amniotic epithelial stem cells in chemotherapy-induced ovarian dysfunction
Source: Inflamm Regen. 2023 Nov 22;43:57. doi: 10.1186/s41232-023-00309-y (PMC10664537; doi:10.1186/s41232-023-00309-y)

**Supporting Information**

**TNF-α and IFN-γ prestimulation enhances the therapeutic efficacy of human amniotic epithelial stem cells in chemotherapy-induced ovarian dysfunction**

Yating Huang^1#^, Qiuwan Zhang^1, 2#^*, Wenjiao Cao^1^, Qinyu Zhang^1^, Lulu Wang^1^, Dongmei Lai*^1, 2^

1. The International Peace Maternity and Child Health Hospital, School of Medicine, Shanghai Jiao Tong University; Shanghai, China 2. Shanghai Key Laboratory of Embryo Original Diseases, Shanghai, China

**^#^** These authors have contributed equally to this work

*Corresponding Author:

Dongmei Lai, MD, PhD

E-mail: [laidongmei@hotmail.com](mailto:laidongmei@hotmail.com)

Qiuwan Zhang, PhD

E-mail:zhangqiuwan@163.com

145, Guang-Yuan Road, Shanghai 200030, P. R. China,

Tel: 86-21-64070434

Fax: +86-21-64074642

**Figure S1. All original full-length gel and blot images in Figure 1.**

Red dashed boxes showed the cropping the gels and blot in Figure 1F and Figure 1H. (N=4 per group)


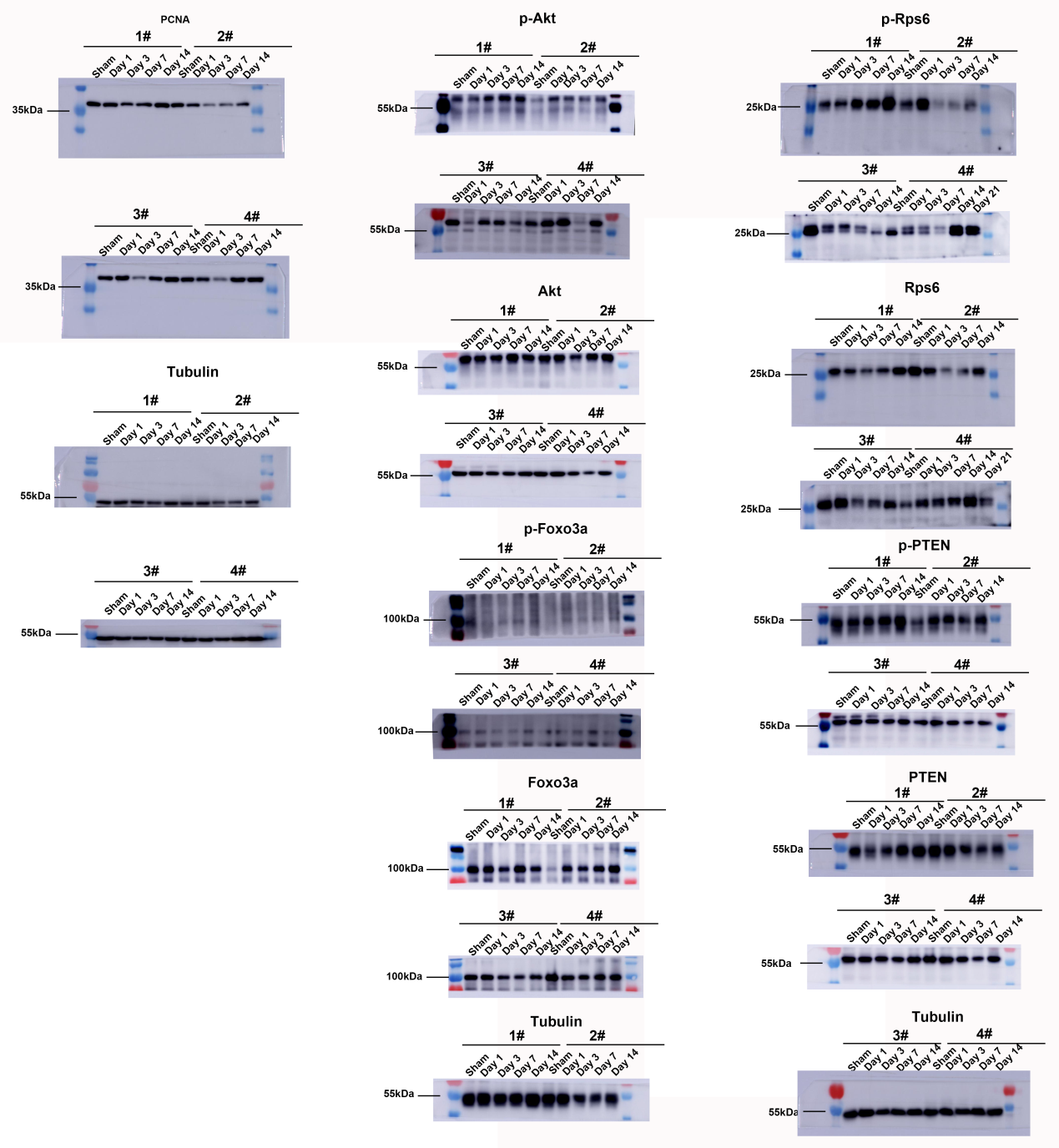


**Figure S2. All original full-length gel and blot images in Figure 3.**

Red dashed boxes displayed the cropping the gels and blot in Figure 3A. (N=4 per group)


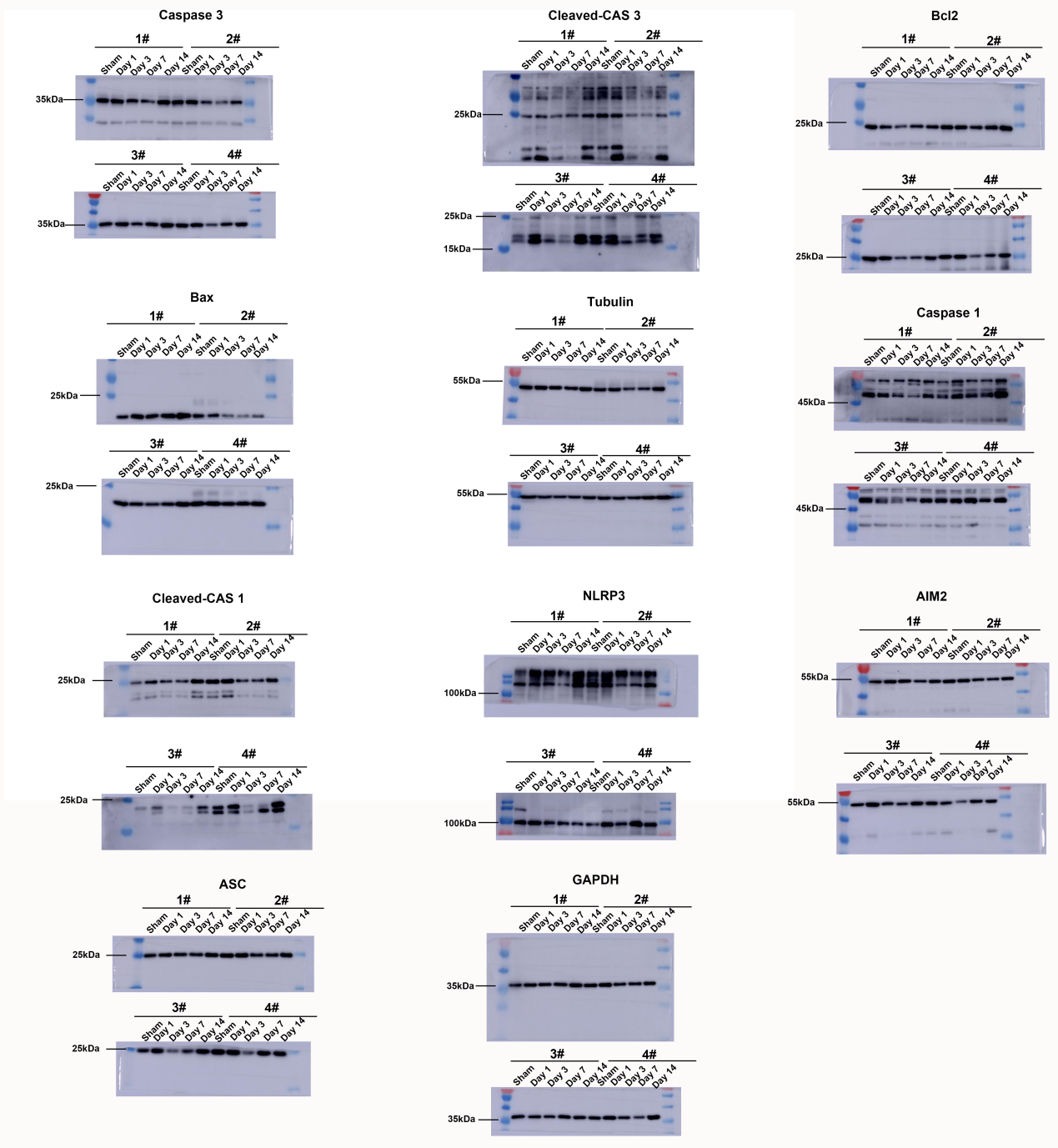


**Figure S3. All original full-length gel and blot images in Figure 5.**

Red dashed boxes displayed the cropping the gels and blot in Figure 5K. (N=4 per group)


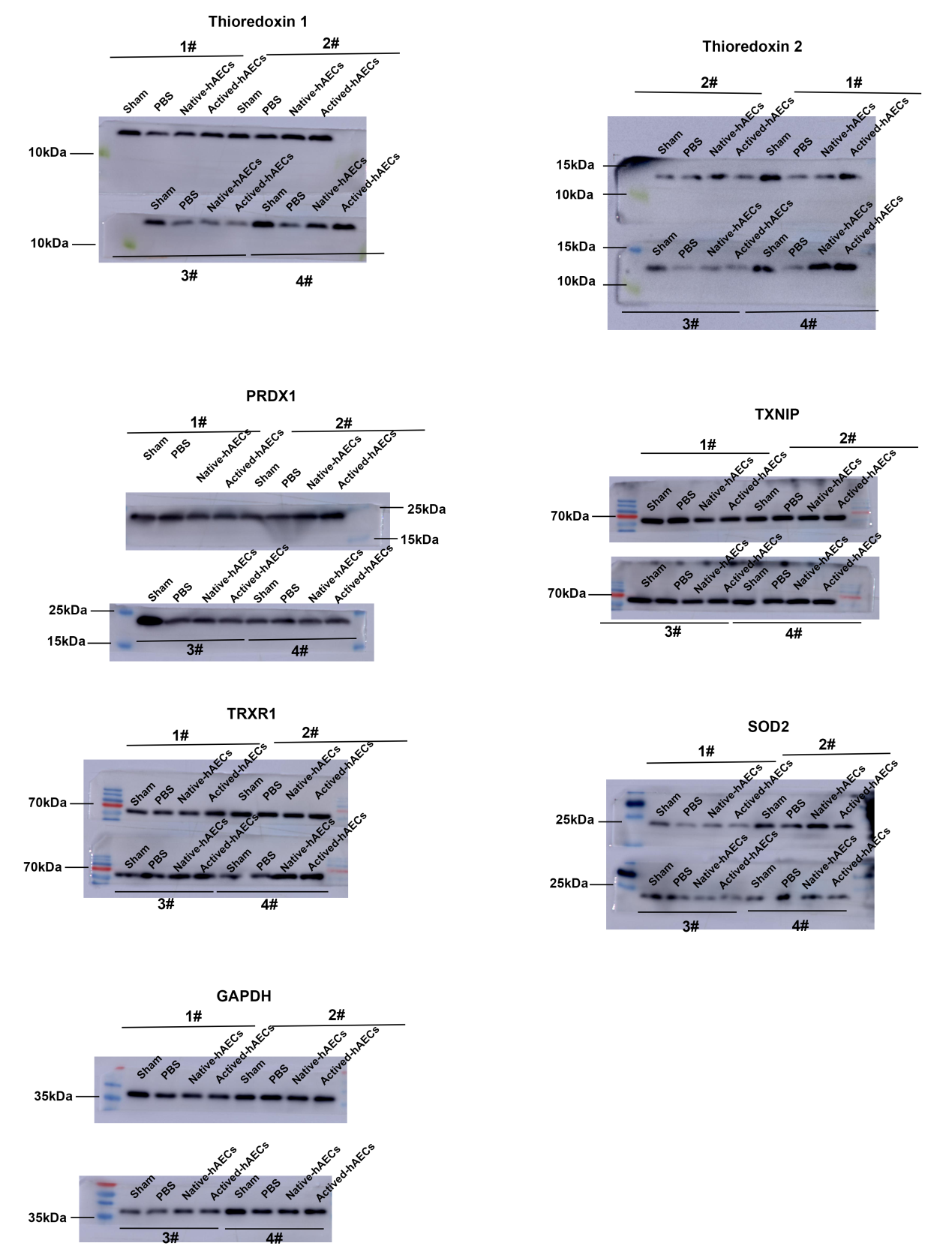

Supplement: Supplementary file 1 — Additional file 1. [file 41232_2023_309_MOESM1_ESM.docx]
